# Analysis of gear surface morphology based on gray level co-occurrence matrix and fractal dimension

**DOI:** 10.1371/journal.pone.0223825

**Published:** 2019-10-22

**Authors:** Bo Wei, Xiaofang Zhao, Long Wang, Bin Hu, Lei Yu, Hongwei Tang

**Affiliations:** 1 Institute of Computing Technology, Chinese Academy of Sciences, Beijing, China; 2 The First Research Institute of the Ministry of Public Security, Beijing, China; 3 University of Chinese Academy of Sciences, Beijing, China; 4 Rocket Force University of Engineering, Xi'an, China; Universidad de Almeria, SPAIN

## Abstract

To investigate morphological characteristics and generation mechanism of the machined gears surface, image characteristics of machined surface morphology including profile roughness, fractal and textural characteristics were studied. the change of profile curves for the surface image is subject to the normal probability density function and the W-M function. The orientation angle of surface texture is 0°, the surface profile curves are the smoothest and have the most uniform, regular textures. When the texture orientation is 45° or 135°, the surface profile curves show large fluctuations, while surface image textures present the deepest grooves and are shown to be distributed most irregularly. Additionally, the influence mechanism of different grinding parameters on the morphological characteristics of machined surface was investigated. The quality of machined surfaces increased with the grinding speed while deteriorated with increasing radial, or axial, feed speeds.

## Introduction

Surface morphological characteristics exert a significant influence on the properties of gear surface including microcontact, friction, wear and lubrication behaviours. How to characterise the micromorphological characteristics of rough surface image has been a research focus in evaluating the quality of machined surfaces. The commonly used roughness coefficient fails to reflect the stochastic behaviour and minutiae of the features of rough surface morphologies, therefore, multiple methods, such as grey-level statistics and fractal theory of surface morphological image, have been widely used to describe the characteristics of surface morphology [1–5]. Haralick [6] proposed a grey level co-occurrence matrix (GLCM) and transformed the grey-level information of Landsat images into texture information. Tian *et al*. [7] established a set of new methods for evaluating unconventional rough surfaces of such ceramics was developed by using GLCM and a neural network. The influences of three parameters including step size, greyscale quantisation and direction on the GLCM were investigated to measure the machined surface morphology of Si_3_N_4_ ceramic. Yang *et al*. [8] introduced a new feature extraction method for texture classification application using dual-tree complex wavelet transform and GLCM. Dual-tree complex wavelet transform is performed on the original image to obtain sub-images, and GLCM of each sub-image is calculated and the corresponding statistical values are used to construct the final feature vector. Fractal theory forms the basis for fractal geometry in modern mathematics and suggests that the local characteristics of different factors including morphology, structure, information, function, and energy have a certain similarity or regularity in the spatio-temporal domain. By utilising the fractal dimensions of an image region, the surface morphological characteristics of the image region can be investigated [9,10]. Presently, the estimating method of the fractal dimensions for complex rough surfaces based on three-dimensional spatial information or image colour information [11]. Pentlan *et al*. [12] proposed that there is a corresponding relationship between fractal theory and grey-level image information. Panin *et al*. [13] studied the influence of various methods of obtaining surface images on the calculated value of fractal dimension as a quantitative characteristic of the surface state. It is demonstrated that images obtained both by a scanning electron microscope and by a photocamera are characterized by a noticeable noise level, which alters the behavior of the fractal dimension. Agnieszka *et al*. [14] applied fractal dimension as a measure of surface roughness, to address structure and function of G protein-coupled receptors. Luo *et al*. [15] proposed a new graphical evaluation of micron-scale surface topography reated to fractal dimension, and investigated the effect of wire electric discharge machining process parameters on surface topography. It shown that the pulse-on times is the most dominant factor in affecting the surface texture. In this study, the roughness, fractal characteristic, and texture characteristic, of the machined surfaces image were investigated. The method helps in the acquisition of global information about the morphological characteristics and the forming mechanism of machined surfaces for gears made by form-grinding.

## Materials and methods

The form grinding wheel was fixed in the principal axis of the FANUC BV 75 vertical machining centre and made to rotate at a high velocity and then the gears were fixed using a parallel-jaw vice and installed on a dynamometer. The axial feed was realised by moving the worktable and then the gear surface was subjected to grinding under liquid-based cooling conditions. Sixteen groups of orthogonal experiments were constructed by using three factors and four levels, as shown in Table 1. The form grinding wheel was a novel wheel made of the binding agent of microcrystal corundum ceramics developed by Sinomach Precision Industry Co., Ltd with dimensions of Φ 200 mm × 20 mm× Φ 32 mm. These gear workpieces are made of carburized and quenched 20CrMnTi steel. By applying the levelling image measurement instrument, the surface morphologies of machined gears were magnified 160 times. Moreover, the morphology of machined gear surfaces at 200 times magnification was observed by using an three-dimensional Olympus profilometer. Apart from traditional methods for evaluating roughness, the morphological characteristics of the surface for the workpiece can be also described by means of grey-level image information, texture features and fractal dimensions.

**Table 1:** Parameters of the orthogonal experiments.

**No.**	**1**	**2**	**3**	**4**	**5**	**6**	**7**	**8**
**Grinding speed** ***v***_***s***_ **(m/s)**	35	35	35	35	45	45	45	45
**Radial feed** ***f***_***r***_ **(mm)**	0.05	0.15	0.25	0.35	0.05	0.15	0.25	0.35
**Axial feed rate** ***v***_***w***_ **(mm/min)**	1500	3500	5500	7500	3500	1500	7500	5500
**No.**	**9**	**10**	**11**	**12**	**13**	**14**	**15**	**16**
**Grinding speed** ***v***_***s***_ **(m/s)**	55	55	55	55	60	60	60	60
**Radial feed** ***f***_***r***_ **(mm)**	0.05	0.15	0.25	0.35	0.05	0.15	0.25	0.35
**Axial feed rate** ***v***_***w***_ **(mm/min)**	5500	7500	1500	3500	7500	5500	3500	1500

## Results and discussion

### Profiles characterisation of the machined surface

**Fig 1:**
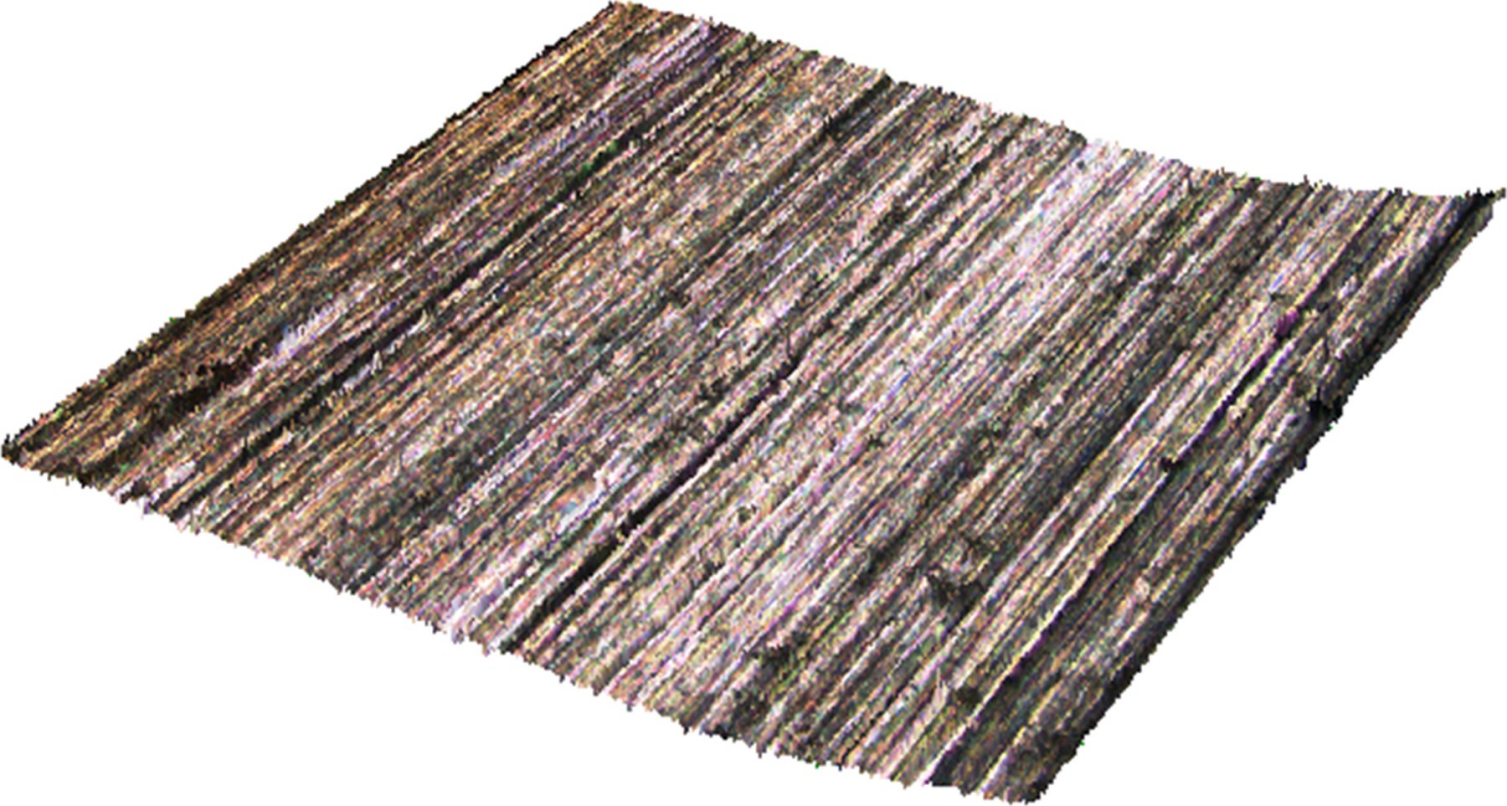
Image of the three-dimensional surface morphology.

The three-dimensional morphology for the machined surface of 20CrMnTi steel gear is shown in Fig 1. The processing parameters used for the surface is: a grinding speed (*v*_*s*_) of 40 m/s, an axial feed rate (*v*_*w*_) of 5,500 mm/min, and a radial feed (*f*_*r*_) of 0.2 mm. Micro-cutting traces and defects in the grinding process arising from abrasive particles on the workpiece surface could be seen. In fact, the grinding process of the workpiece is the accumulated micro-cutting effects simultaneously exerted by large amount of abrasive particles on the workpiece. The grinding processes successively involve scratches, ploughing, and chip formation. Under the effect of ploughing, plastic uplift occurred as the surface materials were pushed to each side. This typical machined surfaces of gears exhibited an approximate arrangement of texture primitives.

The orientation angle of surface texture that is parallel to the direction to the machining trace is defined as 0°, while the surface rotates along the clockwise direction passing through *θ*, the orientation angle of surface texture also seen as *θ*. Based on the experimental parameters of each group, the roughnesses for the machined surface in three groups at four texture orientations of 0°, 15°, 45°, and 90° were stochastically measured.

**Fig 2:**
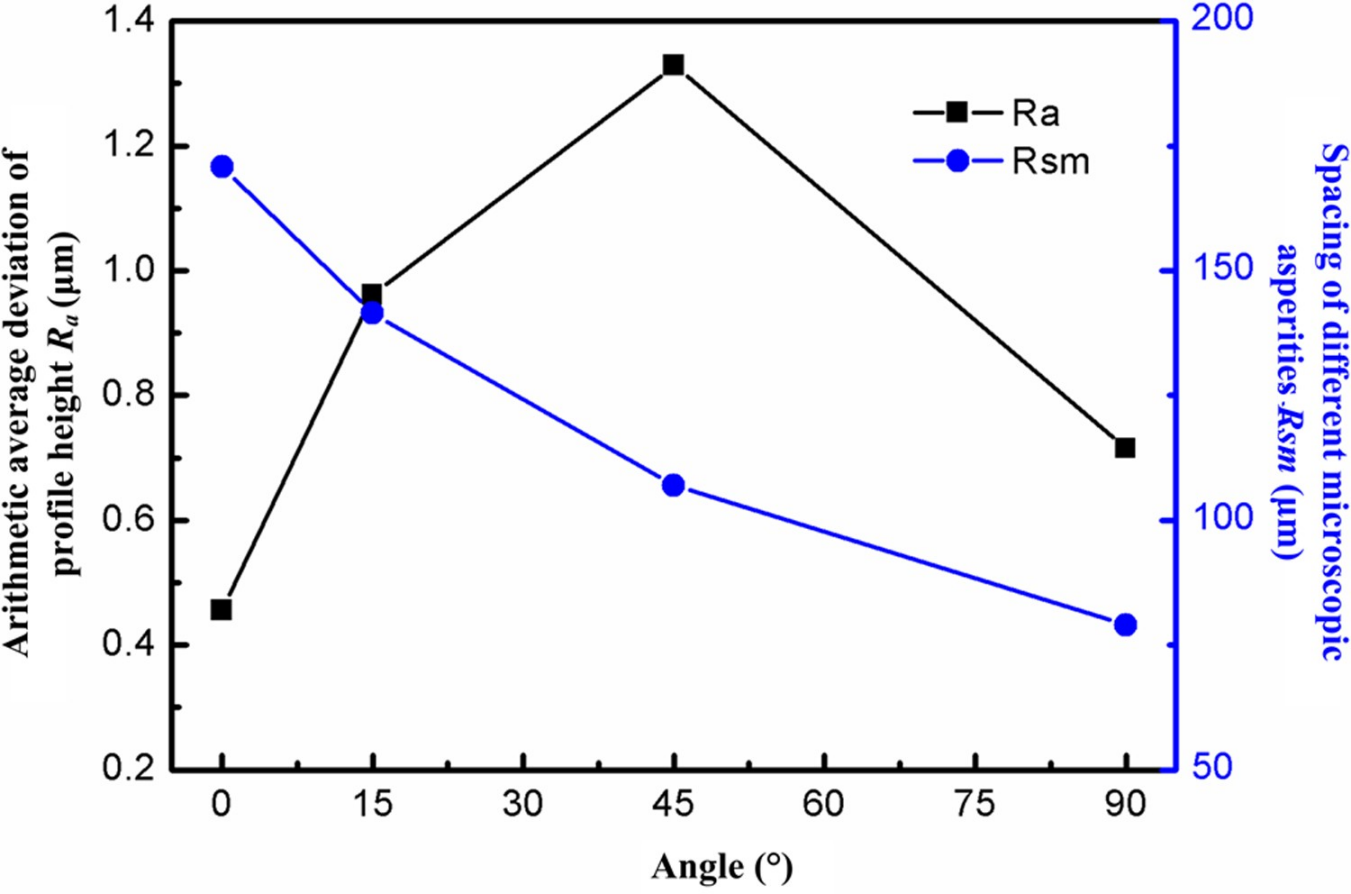
Parameters of microscopic asperity.

**Fig 3:**
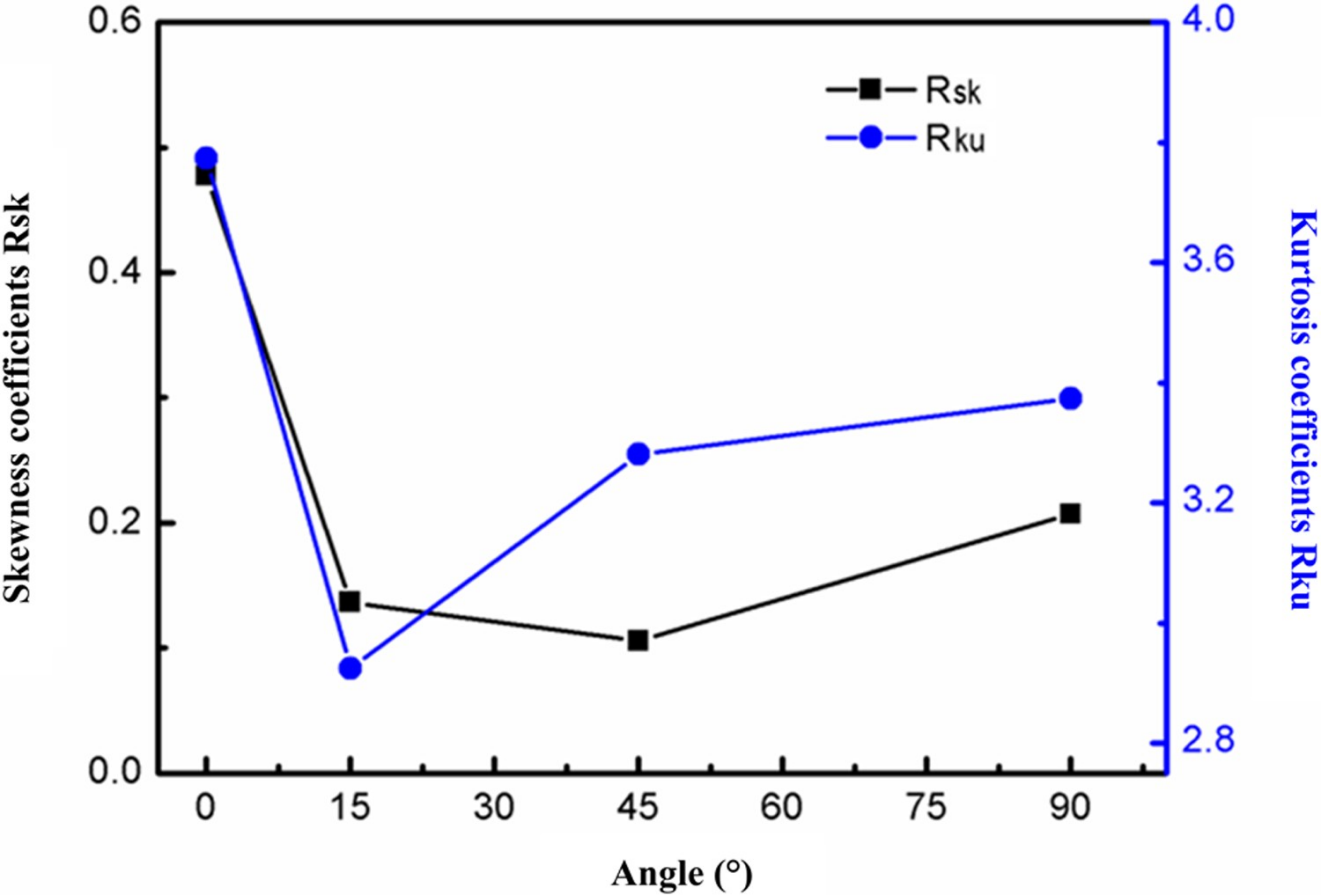
Parameters of profile distribution shapes.

Fig 2 shows the distributions of mean values for two parameters (asperity height *R*_*a*_ (arithmetic average deviation of profile height) and spacing *R*_*sm*_ of different microscopic asperities) at different texture orientations. The characteristic parameters of microscopic asperity height are the smallest at the texture orientation of 0°. As the texture orientation *θ* increased from 0° to 90°, the surface profile curves become increasingly rough, the characteristic parameters of microscopic asperity height reach their maximum at the texture orientation of 45°, and then gradually decline. The spacing *R*_*sm*_ of microscopic asperities reflects the number of distributed wave crests and troughs of the surface profile curves. As *θ* increases from 0° to 90°, the lower the value of *R*_*sm*_, the greater the number of wave crests and troughs on the machined surface in the evaluated wave length, which causes more significant superposition of stresses leading to greater stress concentration. This decreased the fracture strength of a machined surface subjected to applied tensile load. The skewness coefficient *R*_*sk*_ and kurtosis coefficient *R*_*ku*_ are parameters illustrating the distribution shapes of the surface profile curves. The larger the skewness and kurtosis coefficients, the more prone the protruding peaks of the profile curves are to being sharper, indicating a weaker bearing capacity. Fig 3 shows the parameters of distribution shapes of the surface profiles at different texture orientations. As the texture orientation *θ* gradually increases from 0° to 90°, the sharp peak of wave crests of the surface profile declines at first, and then rises. The wave crests of the surface profile at the texture orientations of 0° and 90° exhibited the largest sharp peaks. The mean values of the skewness coefficients *R*_*sk*_ at the four texture orientations all ranged from 0 to 0.4 while those of kurtosis coefficients *R*_*ku*_ ranged from 3 to 4. Therefore, the protruding peak shapes of surface profile curves at different texture orientations mainly appeared as sharp peaks and the stochasticity of distributions of surface profile curves approximately conformed to the normal distribution function.

The heights of the surface profiles along different surface and texture orientation angles of the machined surface exhibited different changes. The measured parameters used to estimate the profiles for the machined surface morphology along different textural orientations are shown in Table 2. When the orientation angle of surface texture is 0°, the longitudinal height of surface profile presents a slow fluctuation. When *R*_*a*_ is 0.6947 um, the microscopic asperity height *R*_*a*_ is the smallest. The profile curves leans towards the upper part of the zero-angle curves with a large transverse span and exhibits the microstructure typical of the surface profile that was shown to be the most sparse. When *R*_*sm*_ is 172 um, the spacing of microscopic asperities, *R*_*sm*_ is the largest. The heights of the surface profile curves at texture orientations of 15° and 45° show large fluctuations, while the value of *R*_*a*_ is at its maximum. The transverse spans of the surface profiles at 15° and 45° decrease compared with that at 0°. The profile curves at the texture orientation of 90° were used to evaluate samples based on this being a standard detection. The profile curves are generally distributed in the two sides of the zero-angle curves while mainly concentrate on the upper part thereof. The longitudinal height of the profile curves exhibits smaller changes than those at 15° and 45° while being larger than that at 0°. The transverse span of the surface profile curves reaches its minimum at *R*_*sm*_ is 79 *u*m, in this case, finest microstructure of profile curves and the spacing of microscopic asperities *R*_*sm*_ is smallest. By analysing the parameters of distribution shapes of profile curves in Table 2, it can be seen that, at texture orientations of 0° and 90°, the skewness coefficient *R*_*sk*_ is greater than 0 while the kurtosis coefficient *R*_ku_ is greater than 3 and the protruding peaks of surface profiles mainly appear as sharp peaks; however, at the texture orientations of 15° and 45°, the skewness coefficients *R*_*sk*_ ≈ 0 while kurtosis coefficients *R*_*ku*_ ≈ 3, while protruding peak of their profiles is mainly seen to be a combination of sharp and blunt peaks.

**Table 2:** Parameters of evaluating the profile shapes of the machined gear.

Angle	*R*_*a*_ (μm)	*R*_*sm*_ (μm)	*R*_*sk*_ (μm)	*R*_*ku*_ (μm)
0°	0.6947	172	0.8881	3.882
15°	0.8996	115	-0.1677	2.9274
45°	1.0573	105.5	-0.0715	2.9714
90°	0.8730	79	0.7144	3.0227

As change in the profile heights of rough surface morphologies is seen as an unstable stochastic process, probability distribution function can be used to describe the distribution and concentration of heights of the protruding peaks on the profile curves. When *R*_*sk*_ is between -1 and 1, and *R*_*ku*_ ranges from 2 to 4, the probability distribution of the height change of profile curves of these rough surfaces is approximately normal. If *a* and *σ* separately refer to the mean value and variance, the probability density function can be expressed as follows [16]:

$$p(z)=\frac{1}{\sqrt{2\pi }\sigma }\exp\left[-\frac{{\left(z-a\right)}^{2}}{2\sigma^{2}}\right]$$ (1)

Table 3 shows the calculation results of the mean value *a* and variance *σ* of the profile curves of the rough surface at four texture orientations of 0°, 15°, 45°, and 90°. The value of *a* reflects the distribution tendency of the location of the primary profile curves. The larger the absolute value of *a*, the further the median line of the arithmetic mean for the profile lies from the zero-angle curve. When *a* = 0, the profile curves are approximately uniformly distributed on the two sides of the zero-angle curve; when *a* > 0, the profile curves are mainly found on the upper part of the zero-angle curve, meanwhile, the larger the mean value, the larger the positive deviation; when *a* < 0, the profile curves are found to distributed on the lower part of the zero-angle curve, while the profile shapes at texture orientations of 0° and 90° are located on the upper part of the zero-angle curve while those for 15° and 45°are distributed on the lower part thereof. The variance *σ* reflects the fluctuations in the height of the rough surface profiles: the larger the variance, the more significant the fluctuations in the profile curves, and the larger the height of the microscopic asperities. The variance, at 45°, was maximised, implying that the fluctuation of the height of the profile curves was the most obvious. The variance was minimised at 0°, which indicated that the height fluctuations were moderate.

**Table 3:** The mean values and variances of the profile curves at four orientations.

Angle	Mean value *a*	Variance σ
0°	0.05	0.60
15°	-0.12	2.99
45°	-0.01	3.37
90°	0.11	2.66

Traditional parameters for characterising surface roughness are affected by both the dimensions and resolution, thus having a certain limitation in the analysis of surface morphology. The partial and overall profile curves of the machined surface morphology of the gears exhibited a certain self-similarity and self-affine characteristics. Existing research shows that fractal theory can be used to characterise the rough surface morphology of workpieces so as to establish the W-M function simulation model for surface profiles. The W-M function shows characteristics including continuity and self-affine characteristics. For the stochastic surface conforming to the normal distribution, *w* = 1.5^*n*^. Since the sampling length and resolution ratio are 800 um and 5/7, respectively, so 1/800 < *w* < 7/10 (namely, -16 ≤ *n* ≤ -1). If *x*, *D*, and *C* refer to the survey coordinate, fractal dimension, and the coefficient of characteristic scale, the W-M function for simulating the rough surface profile can be expressed as follows [17]:

$$z(x)=C^{D-1}\sum_{n=-16}^{-1}\frac{\cos\left(2\pi \cdot 1.5^{n}x\right)}{1.5^{(2-D)n}}$$ (2)

Coefficients *D* and *C* are closely associated with the surface roughness. By employing the box-counting method, the fractal dimensions, scale coefficients and the correlation test of profile curves for the rough surface morphologies at four texture orientations of 0°, 15°, 45°, and 90°were calculated (Fig 1 and Table 4). It can be seen from the table that as the angle gradually increases from 0° to 90°, the finer the microstructure of the surface profile, the larger the corresponding fractal dimensions *D*. As *R*_*a*_ (the height of microscopic asperities) of the profile curves increases, that is, the larger the dimension of profiles, characteristic scale coefficient *C* also increases. The results of the correlation test reveal that the correlations *corr* at the four orientations of 0°, 15°, 45°, and 90° all exceed 99%, implying that the profile curves of the rough surfaces had significant fractal characteristics.

**Table 4:** Fractal characteristics of profile curves at four orientations.

Angle	Fractal dimensions *D*	Characteristic scale coefficient *C*	W-M function scale *C*^*D-1*^	Correlations *cor*
0°	1.2803	2.2987	1.2628	0.9974
15°	1.3203	2.8091	1.3921	0.9975
45°	1.3433	3.4199	1.5252	0.9974
90°	1.3614	2.3796	1.3679	0.9984

### Texture characteristics of the machined surfaces

Gray level co-occurrence matrix (GLCM) uses second-order statistics to measure the gray level variation of image, and reflects the spatial distribution characteristics of gray level of texture. The different morphological features of each different gray-scale image are formed by the repeated changes of gray-scale distribution in spatial position[18–19].Wang *et al*. [7]found that only some characteristic parameters (including contrast, inverse difference moment (IDM), correlation, and entropy) show low correlation and the method is able to acquire a high-accuracy textural classification precision. The test results showed that the change in contrast is generally consistent with that of entropy while the changes in energy and correlation are in agreement with that of IDM. Therefore, only contrast and IDM are used to describe the texture characteristics. In which, contrast (*CON*) reflects the depth of grooves on the surface while *IDM* shows the uniformity and homogeneity of the texture. If *P*(*i*,*j*) refers to the extracted grey level co-occurrence matrix, the contrast ratio and IDM are calculated as follows [15]:

$$CON=\sum_{i}\sum_{j}{\left(i-j\right)}^{2}P(i,j)$$ (3)

$$IDM=\sum_{i}\sum_{j}\frac{P(i,j)}{1+{\left(i-j\right)}^{2}}$$ (4)

**Fig 4:**
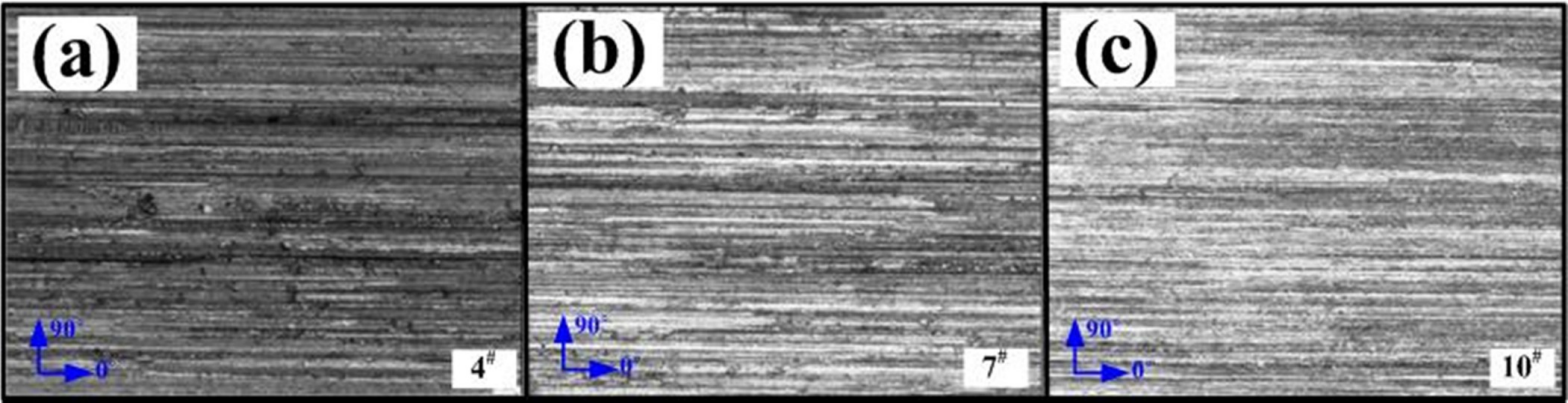
Grey level images of the machined surfaces of gears.

The optical images of morphology for the machined surface with 160 ×magnification are collected. Using MATLAB™ software, a grey level co-occurrence matrix with a compressed grey level of 160 and a step size of 5 is adopted. The changes in the mean values of contrast ratio and IDM for three grey level images at four texture orientations (0°, 15°, 45°, and 90°) in Fig 4 are obtained.

**Fig 5:**
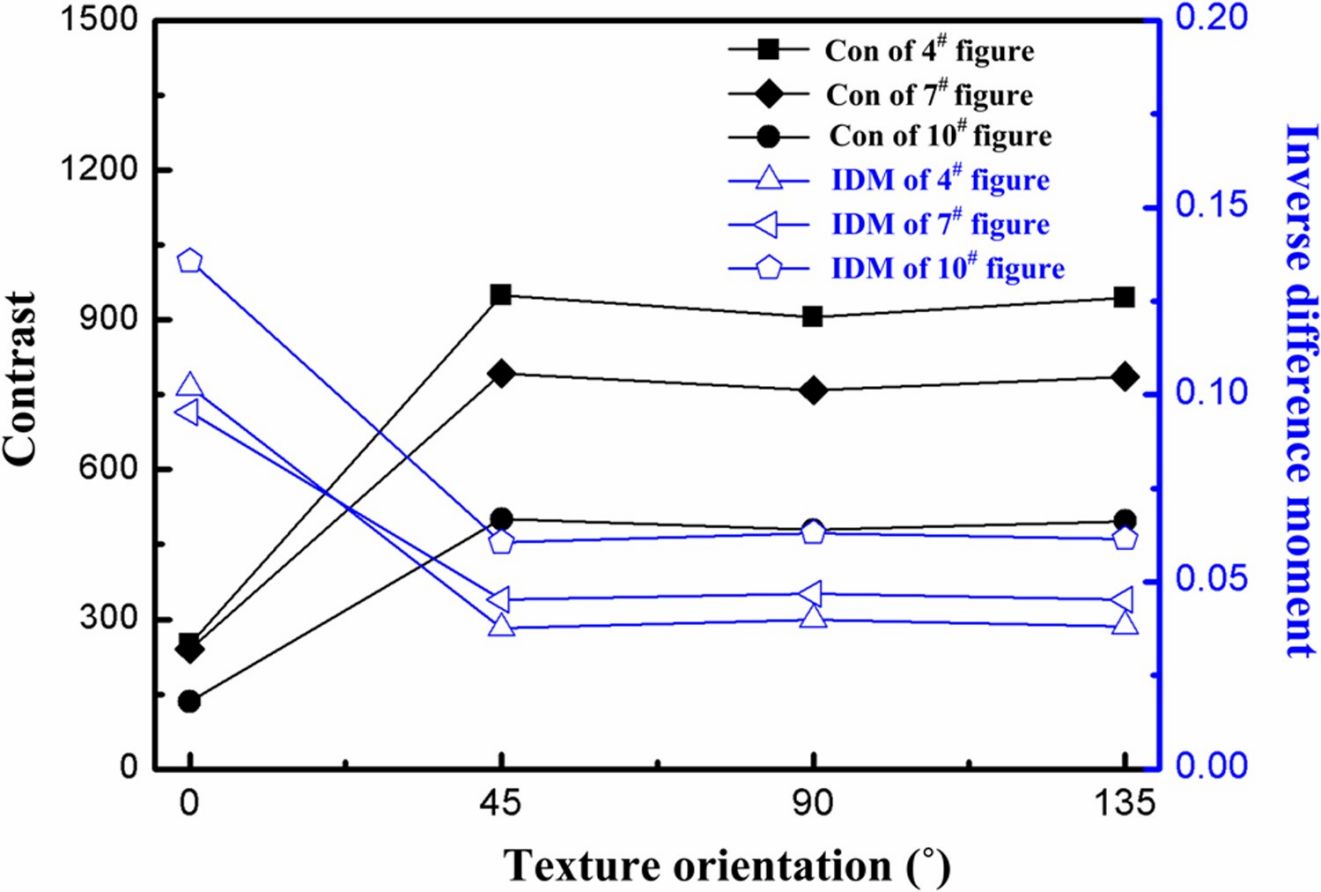
Texture characteristics at different texture orientations.

Fig 5 shows the changes of contrast and IDM with change of texture orientations. At the texture orientation of 0°, the contrast, the height of the grooves on the surface, and the height of the microscopic asperities are all minimised; moreover, the IDM is maximised, a local grey level change is seen to be slight, and surface textures are distributed most uniformly with a regular arrangement thereof. The texture is the most regular and uniform at 0° and that is parallel to the direction of the machining trace. At orientations of 45° or 135°, the contrast ratio, depth of grooves, and the height of surface microscopic asperities are maximised: however, the IDM is minimised, while textures change most regularly evincing the poorest uniformity. Therefore, changes of texture primitives on the machined surface at different texture orientations also differ. As the angle *θ* gradually increases from 0° to 45°, the groove depth, uniformity and regularity of the surface textures gradually increase and reach a maximum at 45°. Afterwards, they were slightly decreased at the texture orientation of 90° and were near-vertical to the direction of the machining trace.

As an essential inherent attribute of images, texture characteristics are able to show the local micromorphology and the attributes of local spatial distribution for textures that approximately show a regular arrangement. Three images of the surfaces of the machined gears based on the machined parameters in each of the four groups were stochastically collected. To guarantee invariance under image rotation, the mean values of texture characteristics at four orientations of 0°, 15°, 45°, and 90° are calculated based on the step size of 5 and a compressed grey level of 60 to describe the texture state of the machined gear surfaces. To investigate the uniformity of image grains and depth of grooves on the machined surface, the results of IDM and contrast are calculated based on the grey level co-occurrence matrix (Table 5).

**Table 5:** Characteristic parameters of the surface textures.

**No.**	**1**	**2**	**3**	**4**	**5**	**6**	**7**	**8**
**Inverse difference moment**	0.0989	0.0841	0.0759	0.0692	0.0973	0.1088	0.0884	0.0876
**Contrast**	285.7	528.6	596.4	768.9	255.5	336.2	591.4	690.6
**No.**	**9**	**10**	**11**	**12**	**13**	**14**	**15**	**16**
**Inverse difference moment**	0.1059	0.0955	0.1255	0.1069	0.1117	0.1161	0.1187	0.1263
**Contrast**	242.0	432.4	349.6	561.6	235.3	349.0	361.2	453.1

**Fig 6:**
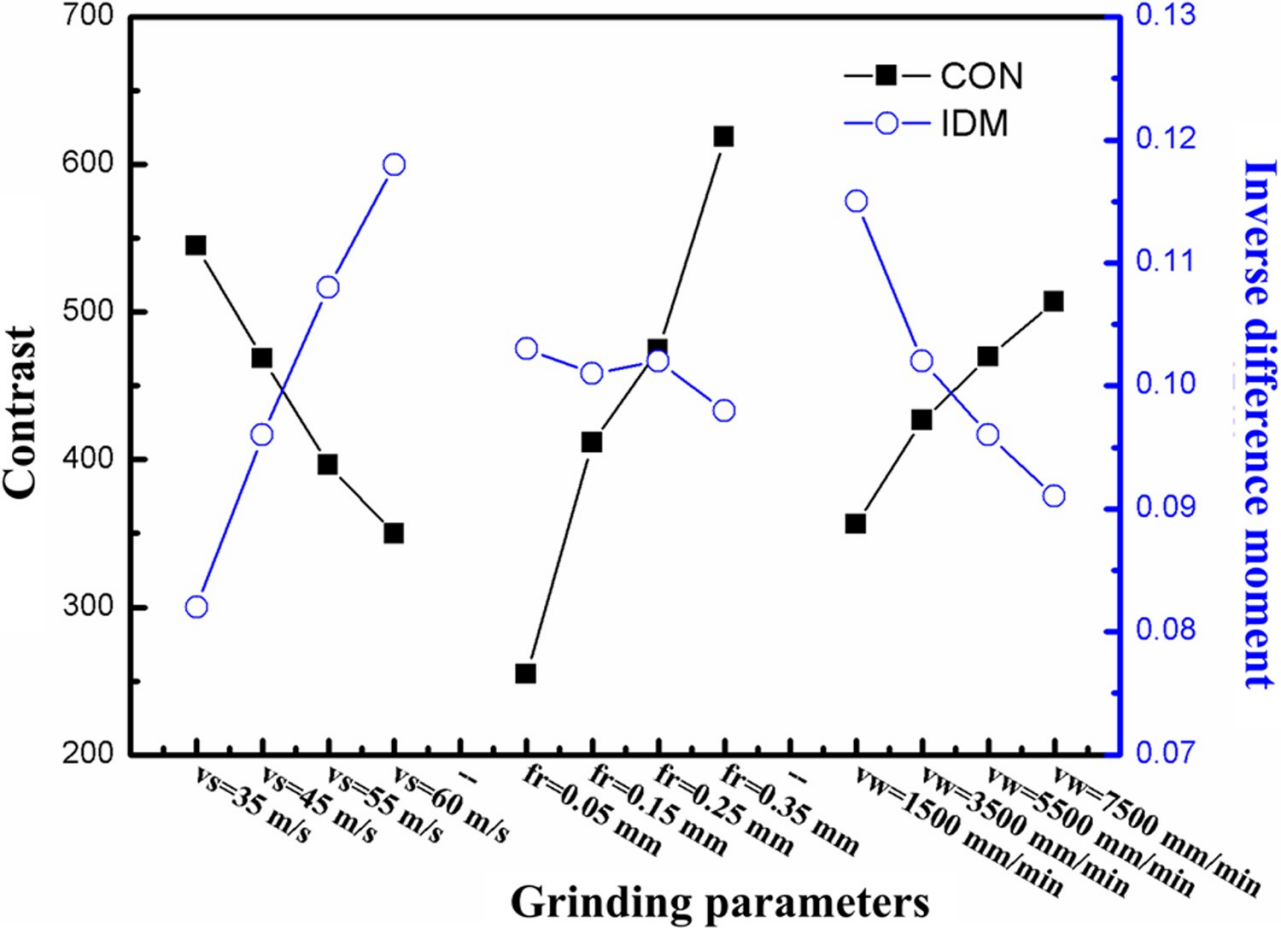
Changes in contrast ratios and inverse difference matrices with grinding parameters.

Variance analysis of the different factors influencing the texture characteristic of the machined surface was conducted: the results indicated that *F* ratios of the grinding speed, radial, and axial, feed rates to IDM were 2.25, 0, and 0.75, respectively. This suggests that the grinding speed exerts the most significant effect on IDM and the uniformity of the machined surface morphology. The *F* ratios of the grinding speed, radial and axial feed rates to contrast are 0.639, 1.994, and 0.367, respectively. This implies that the radial feed has the most significant influence on the contrast and the depth of grooves on the machined surfaces. The changes in the mean values of two texture characteristics under different grinding parameters and horizontal conditions can be intuitively analysed, as shown in Fig 6. As the grinding speed increases, the IDM becomes larger while contrast reduces. In the meantime, the homogeneity and uniformity of the surface textures are increasingly improved and the depth of the grooves on the surface gradually reduces, leading to a smoother surface. This is because, with a faster grinding speed, the number of abrasive particles in the grinding process shows multiplicative growth per unit time. Therefore, the workpiece materials, for the same part, are prone to be subjected to more instances of micro-cutting effects, which result in the reduced thickness of the maximum number of non-deformed chips, and thus is conducive to improving the surface quality. As the radial, or axial, feed rates increase, the IDM gradually reduces and the uniformity and consistency of the surfaces gradually worsen with obvious stochasticity and complexity arising thereon; however, under the same conditions, the contrast gradually increases to cause the depth of grooves on the machined surface to gradually enlarge and local fluctuations of the surface profile increase more significantly in amplitude. The reason for this was that, with increasing radial, or axial, feed rates, the amount of the material removed per unit time increased and the maximum thickness of those undeformed chips also increased; additionally, the uplift induced by plastic deformation was more significant and more residual materials were found on the surface, which reduced the surface machining quality.

**Fig 7:**
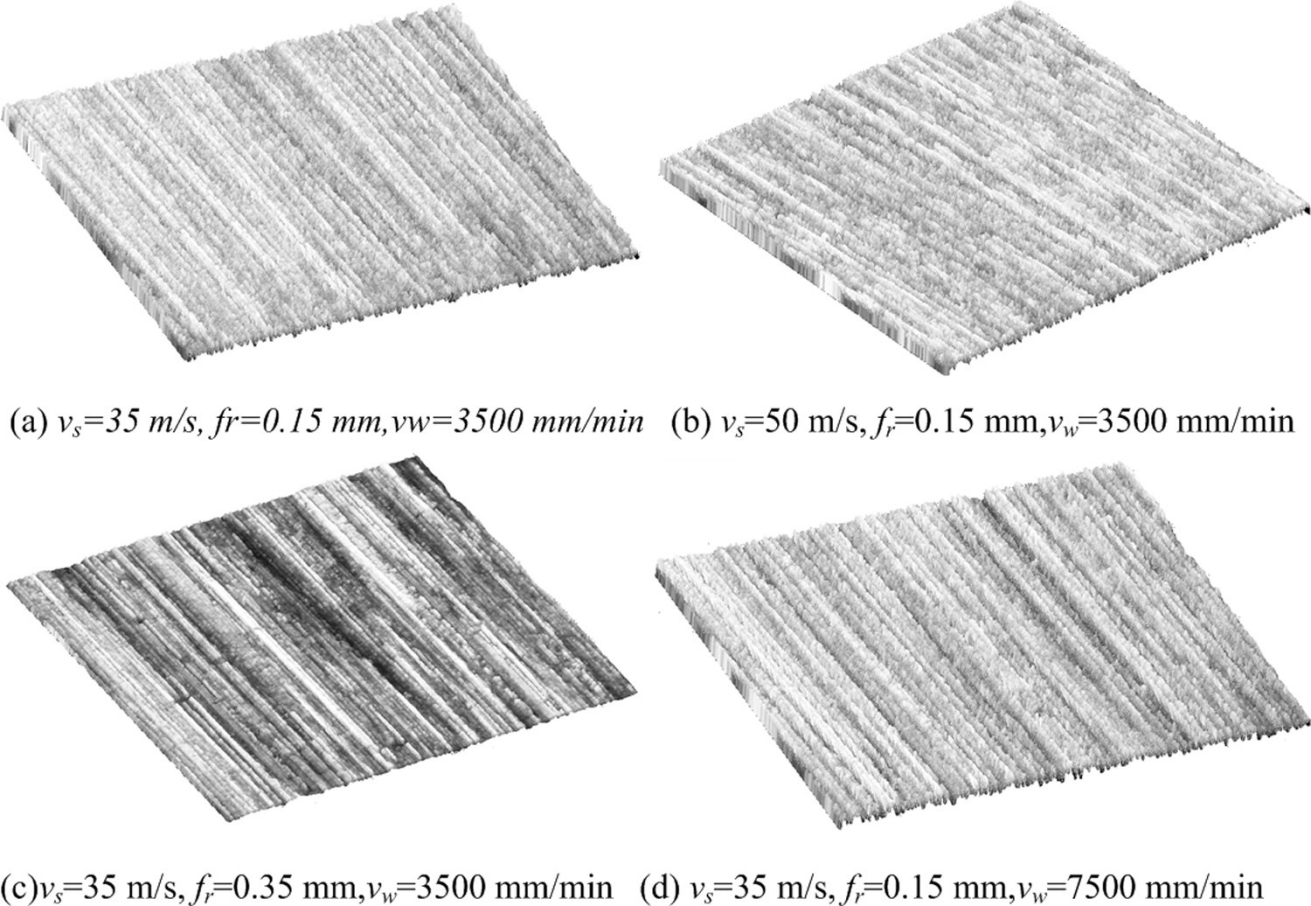
Three-dimensional micro-topography reconstruction based on gray image.

There is a corresponding relationship between the spatial information of grinding surface and the gray information of image. The three-dimensional grinding surface morphology is reconstructed based on the spatial gray distribution, as shown in Fig 7. Comparing the surface characteristics of Fig 7(A) and Fig 7(B), with the increase of grinding speed, the plastic bulges phenomenon weakens, which makes the grinding surface texture more regular and uniform, and is conducive to improving the surface quality. Comparing the surface characteristics of Fig 7(A) and Fig 7(C), with the increase of grinding depth, plastic bulges is more easily formed, which makes the surface texture more rough and sparse, and the deeper the texture groove, the more uneven and irregular. Comparing the surface characteristics of Fig 7(A) and Fig 7(D), the greater the axial feed speed, the more plastic bulges on the grinding surface, the thinner the surface texture, and the greater the degree of non-uniformity.

### Fractal characteristics of the machined surfaces

The morphologies of the machined surface of workpieces show stochastic spatial distribution characteristics, which all exert an important effect on their tribological characteristics including contact mechanics, abrasive resistance, and fatigue resistance performance. The spatial information of the machined surfaces had a corresponding correlation with its grey level image information. If the grey level image with *M × M* pixels corresponds to grey level *Z* at point (*x*,*y*) on a coordinate plane, the grey level image can be seen as a curved face *Z* = *f*(*x*,*y*) in three-dimensional space. Fractal dimensions are an important measure used for describing the irregularity, effective spatial occupation, and complexity of complex morphologies of a machined surface. By employing a differential box-counting approach, the fractal dimension of machined surfaces of the gears can be calculated. The effect of this three-dimensional space covering estimation approach is similar to that involving the covering of irregular curves by using two-dimensional square grids. MATLAB™ was programmed using the differential box-counting approach so as to extract the values of fractal dimensions of grey-level optical images for the machined gear surfaces. The calculated fractal dimensions and the detected surface roughnesses for machined surfaces of the formed grinding wheel are shown in Table 6.

**Table 6:** Fractal dimensions and surface roughness of the machined surfaces.

**No.**	**1**	**2**	**3**	**4**	**5**	**6**	**7**	**8**
**Fractal dimension *D***	2.3570	2.3880	2.3991	2.4493	2.3675	2.3323	2.3903	2.3966
**Surface roughness *R***_***a***_ (**μm)**	0.75	0.96	1.21	1.35	0.68	0.79	1.01	1.16
**No.**	**9**	**10**	**11**	**12**	**13**	**14**	**15**	**16**
**Fractal dimension *D***	2.3635	2.3875	2.3135	2.3813	2.3669	2.3707	2.3616	2.3294
**Surface roughness *R***_***a***_ **(μm)**	0.7	0.85	0.76	0.88	0.73	0.76	0.78	0.77

**Fig 8:**
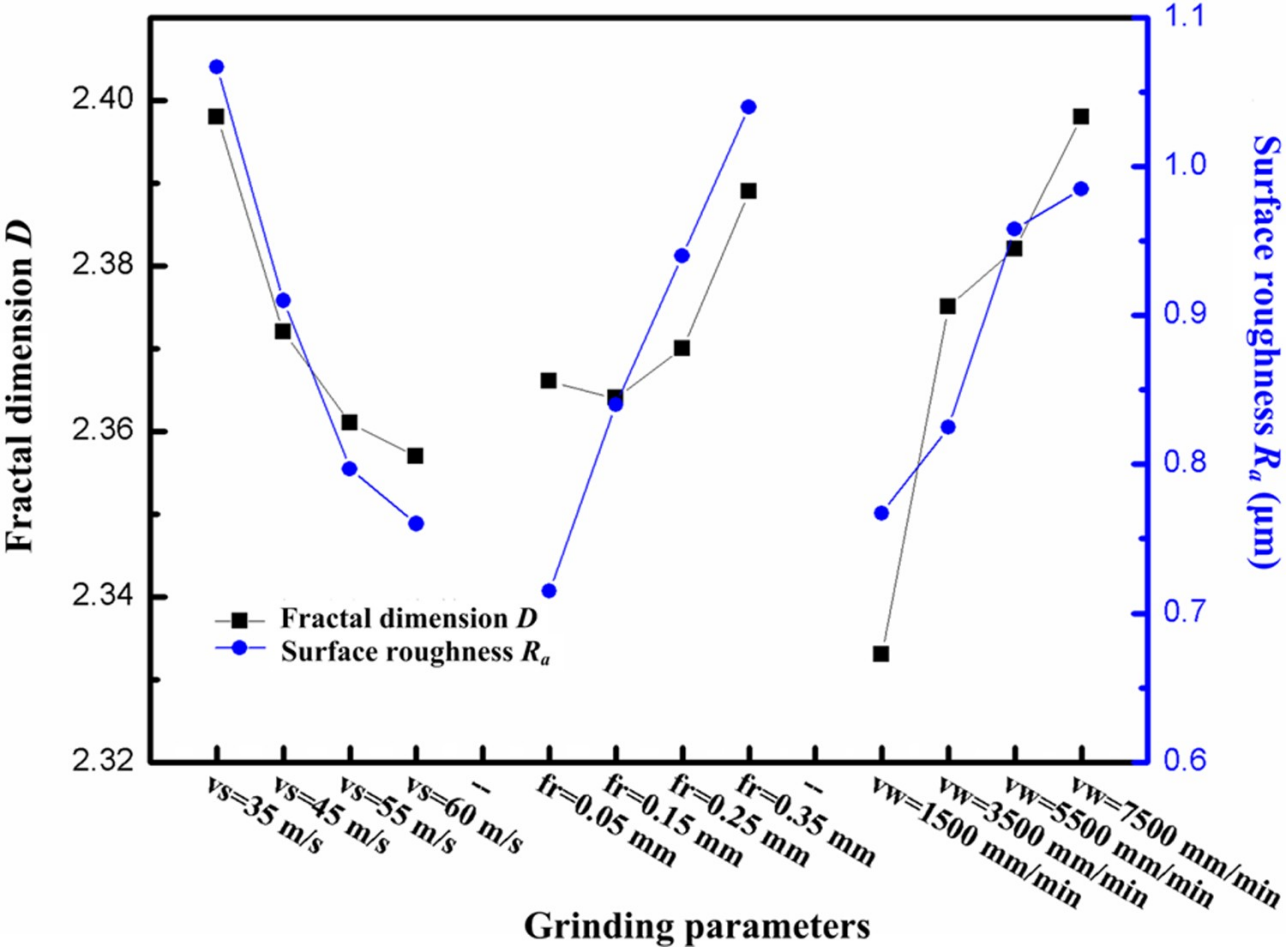
Changes in fractal dimensions and roughness with grinding parameters.

The variance ratios *F* of different influence factors on the surface fractal dimensions *D* and the surface roughnesses of the surface *R*_*a*_ were analysed. The results showed that the influence of the axial feed rate, grinding speed, and radial feed rate on fractal dimension *D* reduce, in descending order, and their variance ratios *F* are 1.8, 0.8, and 0.4, respectively: however, the influences of the axial feed rate, grinding speed, and radial feed rate on surface roughness *R*_*a*_ grow in increasing order, and the variance ratios of the three parameters are 0.664, 1.06, and 1.176, respectively. Therefore, the axial feed rate exerts the most significant influence on the irregularity of the surface morphology while the radial feed rate exerts the greatest effect on the height of microscopic asperities on the surface. Under different horizontal conditions with multiple grinding parameters, the change in the mean values of the surface fractal dimension *D* and surface roughness *R*_*a*_ are calculated, based on which, the curves pertinent to the effects of the different parameters are obtained by intuitive analysis (Fig 8). The values of *D* and *R*_*a*_ both decrease with increasing grinding speed and increased with increasing radial, and axial, feed rates. This is because, with increased grinding speed, the number of times the workpiece surface undergoes abrasion per unit time increases geometrically. The smoother the surface morphology of the workpieces, the more uniform, regular, and finer the texture thereon. With increasing radial, and axial, feed rates, the material removal rate per unit time increases and the uplifting induced by plastic deformation became more significant. Moreover, the more residual materials that have not been removed by grinding, the more rough, complex, and irregular the surface morphology.

## Conclusions

Image characteristics of the gear surface morphology machined form-grinding wheels were investigated to assess the influence of different grinding parameters on their characteristics including profile roughness, fractal characteristics, and textural characteristics. The surface profile curve model was characterised by using normal probability density and W-M functions. As the orientation angle of surface texture increases from 0° to 90°, the most complex surface texture and the largest surface roughness at 45°, and the sharpness of protruding peaks reaches a minimum at 15°. Moreover, the surface fractal dimension, roughness, and contrast all decrease with increasing grinding speed, yet increase with increasing radial, or axial, feed rates. The IDM increases with the grinding speed, and decreases with increasing radial, and axial, feed rates. The grinding speed exerts the most significant influence on the uniformity or homogeneity of the surface morphology while the influence of the radial feed rate on surface irregularity is the most significant. Moreover, the radial feed rate exerts the most significant effect on the height of the microcosmic asperities, or depth of grooves, on the machined surface morphology image.
